# Cross‐flow microfiltration for isolation, selective capture and release of liposarcoma extracellular vesicles

**DOI:** 10.1002/jev2.12062

**Published:** 2021-02-16

**Authors:** Lucia Casadei, Adarsh Choudhury, Patricia Sarchet, Prashanth Mohana Sundaram, Gonzalo Lopez, Danielle Braggio, Gita Balakirsky, Raphael Pollock, Shaurya Prakash

**Affiliations:** ^1^ Comprehensive Cancer Center The Ohio State University Columbus Ohio USA; ^2^ Department of Mechanical and Aerospace Engineering The Ohio State University Columbus Ohio USA

**Keywords:** cross flow filtration, DNA, eEV, extracellular vesicles, lEV, liposarcoma, MDM2, microfluidics, nanofluidics, tangential flow separation

## Abstract

We present a resource‐efficient approach to fabricate and operate a micro‐nanofluidic device that uses cross‐flow filtration to isolate and capture liposarcoma derived extracellular vesicles (EVs). The isolated extracellular vesicles were captured using EV‐specific protein markers to obtain vesicle enriched media, which was then eluted for further analysis. Therefore, the micro‐nanofluidic device integrates the unit operations of size‐based separation with CD63 antibody immunoaffinity‐based capture of extracellular vesicles in the same device to evaluate EV‐cargo content for liposarcoma. The eluted media collected showed ∼76% extracellular vesicle recovery from the liposarcoma cell conditioned media and ∼32% extracellular vesicle recovery from dedifferentiated liposarcoma patient serum when compared against state‐of‐art extracellular vesicle isolation and subsequent quantification by ultracentrifugation. The results reported here also show a five‐fold increase in amount of critical liposarcoma‐relevant extracellular vesicle cargo obtained in 30 min presenting a significant advance over existing state‐of‐art.

## 1 INTRODUCTION

Mesenchymal origin liposarcoma (LPS) is the most common human sarcoma (Bill et al., 2016; Jaques et al., 1990). Despite surgery and adjuvant treatment, more than half the patients develop recurrent or metastatic disease (Anaya et al., 2009). The majority of patients with metastatic soft tissue sarcoma show a median survival rate of < 1 year (Karavasilis et al., 2008). Current diagnostic methods rely on invasive tissue biopsies or are combined with whole or part‐body imaging to identify tumor mass and location (Zhu et al., 2014). LPS poses a clinical challenge due to its anatomical location, with studies showing that tumors found in the retroperitoneum are more likely to recur post‐surgical resection compared to the tumors located at the extremities (Azumi et al., 1987; Fabre‐Guillevin et al., 2006). In addition, the challenges with single tissue biopsies (Aguilar‐Mahecha et al., 2017; Dolgin, 2016), tumor heterogeneities (Arkun et al., 1997), and the potential for distant metastases (10%‐50%) (Tseng et al., 2013) underlie poor prognosis (Hasegawa et al., 2000) and poor overall survival rates (Schwartz et al., 2019). Among the sub‐types of LPS, de‐differentiated liposarcoma (DDLPS) poses a special challenge due to the high rates of recurrence and the higher potential for metastatic disease compared to other LPS sub‐types (Bill et al., 2016; Tseng et al., 2013).

The diagnostic and prognostic challenges prompted us and other researchers to study mechanisms of progression of this disease and to explore the use of circulating biomolecules like micro‐ribonucleic acids (miRNAs) and deoxyribonucleic acid (DNA) that could serve as potential biomarkers for early stage detection and are accessible through bodily fluids (He et al., 2015; Jin et al., 2012). Past work has shown that miR‐25, miR‐92, and *MDM2* DNA may be associated with the development and progression of DDLPS (Casadei & Pollock, 2020; Casadei et al., 2017, 2019). Moreover, it was discovered that these miRNAs and DNA serve as cargo contained within extracellular vesicles (EVs), released from tumors and present in the circulatory system (Casadei & Pollock, 2020; Casadei et al., 2017, 2019).

EVs are a heterogeneous collection of membrane‐bound carriers with complex cargoes including proteins, lipids, miRNAs and nucleic acids (Maas et al., 2017; Raposo & Stoorvogel, 2013). There has been significant debate on the nomenclature for these membrane‐bound carriers, which are observed as sub‐micron diameter particles. The Society for Extracellular Vesicles recently suggested standardizing the nomenclature to designate a particle as an extracellular vesicle (Russell et al., 2019). We distinguish two main subtypes of EVs i.e., small EVs (sEVs) 30–150 nm in diameter and large EVs (lEVs) 100–1000 nm in diameter.

Often, EV‐contained cargo presents a snapshot of the host cell (Simons & Raposo, 2009; Van Niel et al., 2006); however, the composition of EVs can be different from the cells of origin due to selective cargo sorting (Głuszko et al., 2019). In oncology, EVs are merging as critical for their ability to transfer information locally within the tumor microenvironment and also to distant tissue sites. Moreover, EVs and contained cargo can also serve as biomarkers retrievable through ‘liquid biopsies’ that facilitate minimally invasive tumor analyses (Whiteside, 2016). Despite the potential diagnostic and prognostic utility, the practical relevance of using EVs for routine analysis is limited mainly because of the time consuming and/or expensive methodologies required for the EVs isolation and due to subsequent low yields of EV‐cargo obtained (Vozel et al., 2017).

Currently, EV isolation by ultracentrifugation is considered the ‘gold standard’ for comparison between methodologies. Ultracentrifugation involves several centrifugation steps at progressively higher spin speeds (He et al., 2014; Momen‐Heravi et al., 2012); however, it fails to separate different EV sub‐types. To avoid co‐isolation of EV sub‐types, studies have reported the utilization of size‐based filtration along with differential centrifugation to sediment larger EVs such as microvesicles and apoptotic bodies (Kalra et al., 2016). Despite these advances, ultracentrifugation yields inconsistencies in EV recovery due to differences in biofluid viscosities, limited recovery of the total EVs (∼5%) (Lamparski et al., 2002; Momen‐Heravi et al., 2013), degradation of EV quality due to disruption of membrane topology, EV aggregation, and decoration of EVs with other sample components (Russell et al., 2019). In addition to ultracentrifugation, precipitation technologies using proprietary kits such as ExoQuick (Yamada et al., 2012) have also been used to capture EVs from biofluids. Although the recovery rates (∼16%) are higher than ultracentrifugation, this method requires longer precipitation times (∼12 h) and is relatively expensive (∼$50/test). Finally, the purity of EVs captured using ExoQuick suffers from low specificity due to co‐precipitation of soluble non‐exosomal proteins (Sidhom et al., 2020). In addition to the clinical and healthcare costs that accompany high infrastructure requirements and reagent costs, the presence of contaminated artifacts further adds on to the challenges of realizing tumor‐derived EVs as a biomarker in clinical settings (Gyorgy et al., 2011; Rood et al., 2010).

Due to these challenges for isolation and capture of EVs, microfluidic systems have attracted attention for their ability to precisely manipulate small liquid volume samples (100 μl‐8 ml) to separate EVs in a relatively short time (10‐200 min) as previously described (Contreras‐Naranjo et al., 2017). However, current microfluidic devices remain limited in application due to complex design and fabrication, insufficient quality of captured EVs, lower purity extraction of EV cargo, and co‐isolation of multiple EV sub‐types (Contreras‐Naranjo et al., 2017; Liga et al., 2015).

In this report, we describe a multi‐layer, micro‐nanofluidic device that was used to isolate and capture EVs from both human liposarcoma‐derived Lipo246 cell line conditioned media (LCCM) and DDLPS patient serum. The device integrated the unit operations of both size‐based separation (Cho et al., 2016; Davies et al., 2012; Dehghani et al., 2019) using microfiltration and immunoaffinity‐based capture of EVs (Chen et al., 2010; Hisey et al., 2018; Kanwar et al., 2014) within the same device. Post‐capture, the EVs were eluted for subsequent off‐device analysis to verify integrity of the EV‐cargo. The micro‐nanofluidic device was operated in a cross‐flow filtration configuration with size‐based microfiltration achieved through a nanocapillary array membrane (NCAM) (Rangharajan et al., 2016) with 200 nm nominal capillary diameter allowing EVs with nominal size < 200 nm to pass through the NCAM for capture. Since CD63 is generally considered to be a specific marker of endosome‐derived EVs (such as sEV) (Russell et al., 2019), the anti‐CD63 antibody was used to functionalize the separation channel. This device enabled ∼32% recovery of EVs present from DDLPS patient blood serum. Moreover, ∼76% of EVs present in the LCCM were recovered in less than 1 hour from 300 μl sample volume compared to ultracentrifugation‐based EV separations requiring an isolation time of approximately 4 h with a total sample volume of 15 ml.

## 2 MATERIALS AND METHODS

### 2.1 Cell culture

Human LPS cell lines Lipo246 were previously established in our laboratory (Peng et al., 2011). Cells were maintained using standard conditions and were grown in DMEM (Gibco), supplemented with 10% (vol/vol) FBS. For EVs production and LCCM collection, cells were serum‐starved (treated with serum‐free DMEM) for 48 h. In preparation for use in the microfluidic device, LCCM was centrifuged at 2000 g for 20 min.

### 2.2 Patients and clinical samples

Blood samples of LPS patients were collected at The Ohio State University James Cancer Medical Center. Written informed consent was received from participants prior to inclusion in the study, in accordance with the Helsinki Declaration under the auspices of a protocol approved by the Ohio State University Wexner Medical Center Institutional Review Board. Patient venous blood was collected in Vacutainer Plus whole blood tubes with K2 EDTA (BD). Blood serum was retrieved from the whole blood samples *via* centrifugation at 1,900 g for 10 min at 4°C, then aliquoted and stored at ‐80°C until analysis. In this work, sera from 5 patients were used to quantify performance and show proof‐of‐concept operation of the micro‐nanofluidic device for actual patient samples. In preparation for use in the microfluidic device, the serum was centrifuged at 3000 g for 15 min at 4C to remove cells and debris.

### 2.3 Device fabrication

The fabrication and broad operation of the micro‐nanofluidic device for the isolation, capture, and elution of LPS EVs has been reported previously (Kuo et al., 2003; Mohana Sundaram et al., 2020). Briefly, the micro‐nanofluidic device was fabricated using polydimethylsiloxane (PDMS) with soft lithography (Prakash & Yeom, 2014). The microchannel features (3 cm (L) x 500 μm (W) x 150 μm (H)) were patterned on a n‐type 4″ silicon wafer (University Wafers, USA) using SU‐8 2050 (MicroChem) and exposed with ultraviolet light (EV Group, Model: EVG 620 contact aligner) to yield the molds to cast the PDMS. Silicone elastomer base and curing agent (Ellsworth Adhesives) were mixed in a 10:1 ratio, de‐gassed and poured on the above prepared silicon master, degassed again, and cured at 65°C for 5 h. The cured PDMS was peeled from the silicon master and cut into individual devices. Next, to access the injection channel and the separation channel, through‐holes were punched with 1.5 mm biopsy punches on the PDMS with the injection channel.

**FIGURE 1:**
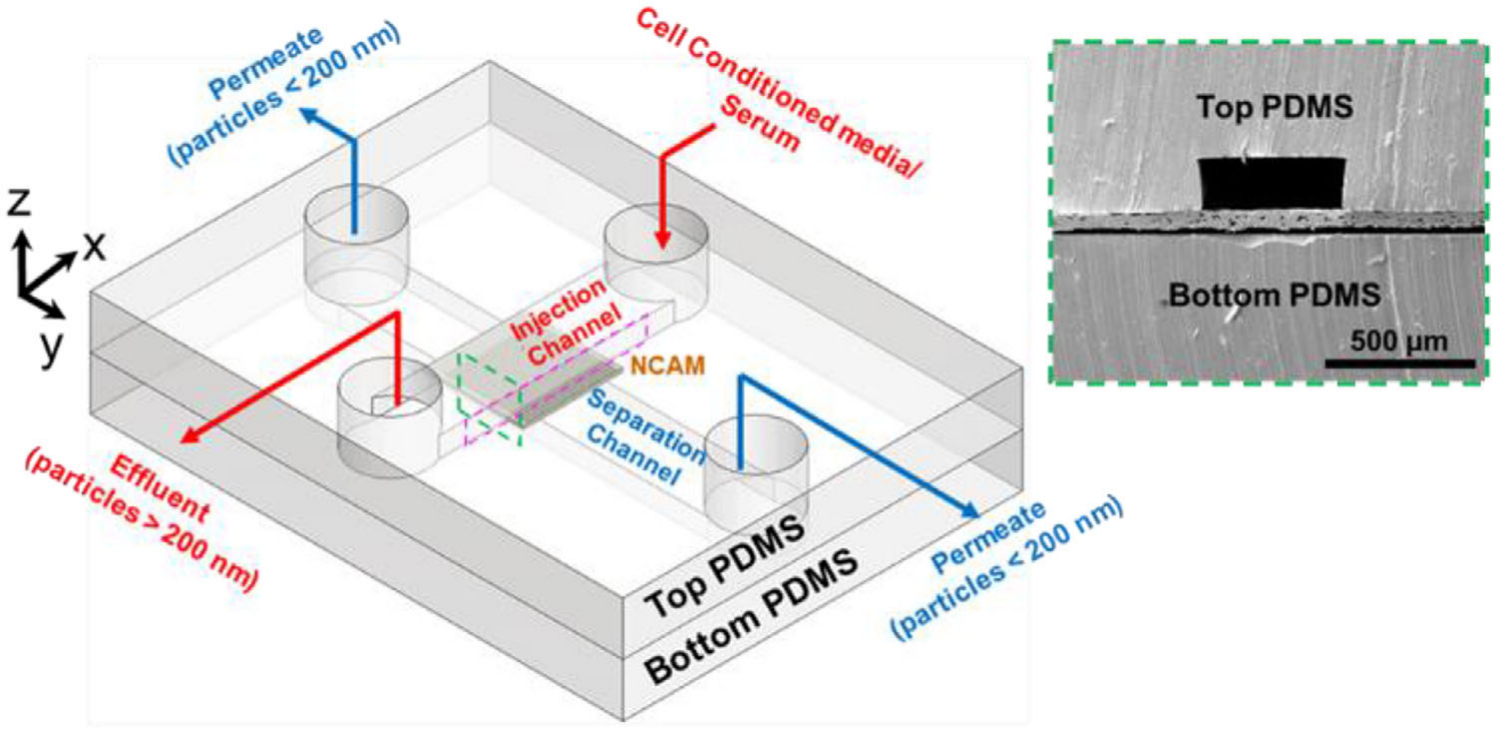
Schematic of the microfluidic channels (each 500 μm wide and 150 μm high) separated by a nanocapillary array membrane (NCAM; nominal capillary diameter = 200 nm; pore density = 3×108 pores/cm^2^; membrane size = 5 mm x 5 mm) (light grey between top and bottom PDMS). The green dotted line represents the cross‐section that was utilized for scanning electron microscopy (SEM) characterization of the device. The inset shows representative images of SEM image showing cross‐section of the microfluidic channel and the NCAM. The pink dotted line represents the x‐z plane view of the COMSOL geometry as seen in Figure 3A

The nanofluidic separator for microfiltration is a polycarbonate track‐etched NCAM (GE Water & Process Technologies) with nominal capillary diameter of 200 nm and a manufacturer reported nanocapillary (or nanopore) density of 3 × 10^8^ pores/cm^2^. Bonding of NCAM to the PDMS was carried out by following a previously reported protocol (Aran et al., 2010). Briefly, a 5 mm x 5 mm NCAM section was oxygen plasma treated at 600 mTorr for 3 min using oxygen plasma chamber (Harrick Plasma, Model: Plasma Cleaner PDC‐001). This was followed by the silanization of the NCAM with a 5% (3‐Aminopropyl) triethoxysilane (APTES; Sigma Aldrich) solution diluted in double distilled water at 80°C. The silanized NCAM and the PDMS monolith with the separation channel were oxygen plasma treated at 600 mTorr for 60 s and bonded to each other (Prashanth et al., 2020). Finally, the PDMS containing the monolith of the injection channel and the NCAM‐separation channel were treated again with oxygen plasma at 600 mTorr for 60 s. The treated surfaces were then bonded with their channels aligned perpendicular to each other by visual inspection (Figure 1).

### 2.4 Separation channel surface modification

Surface functionalization for capturing microfiltered (or isolated) EVs relies on previously reported methods (Mohana Sundaram et al., 2020; Prakash et al., 2009; Rangharajan et al., 2015; Wu et al., 2010). Briefly, the micro‐nanofluidic device was treated with an oxygen plasma at 600 mTorr for 4 min. After plasma treatment, the separation channel was functionalized in a glove bag continuously purged with dry nitrogen (N_2_). 2% APTES solution diluted in 200 proof ethanol (Decon Labs Inc.) was flushed through the separation channel to achieve the surface modification with the APTES solution treating the surface for 2 h (Prakash et al., 2009; Prakash et al., 2007; Rangharajan et al., 2015). Next, 200 proof ethanol was flushed through the separation channel to remove any physiosorbed APTES. Then, a 5% glutaraldehyde solution in 1X phosphate buffer saline (PBS; Corning) was incubated for 30 min in the microchannel followed by treating the channel with 50 μg/ml of primary antibody anti‐CD63 (Ancell) diluted in 1X PBS at 4°C for 8 h. Finally, the separation channel was filled with 1X PBS followed by 50 μl of 1X PBS placed at both ports of the separation channel to avoid drying and the device was stored at 4°C until further use (Hisey et al., 2018). Surface functionalization relies on several previous reports including the use of the anti CD‐63 antibody for immunoaffinity capture of EVs (Long et al., 2006; Prakash & Karacor, 2011; Prakash et al., 2007, 2009; Wu et al., 2010).

### 2.5 Capturing isolated EVs and elution for analysis

**FIGURE 2:**
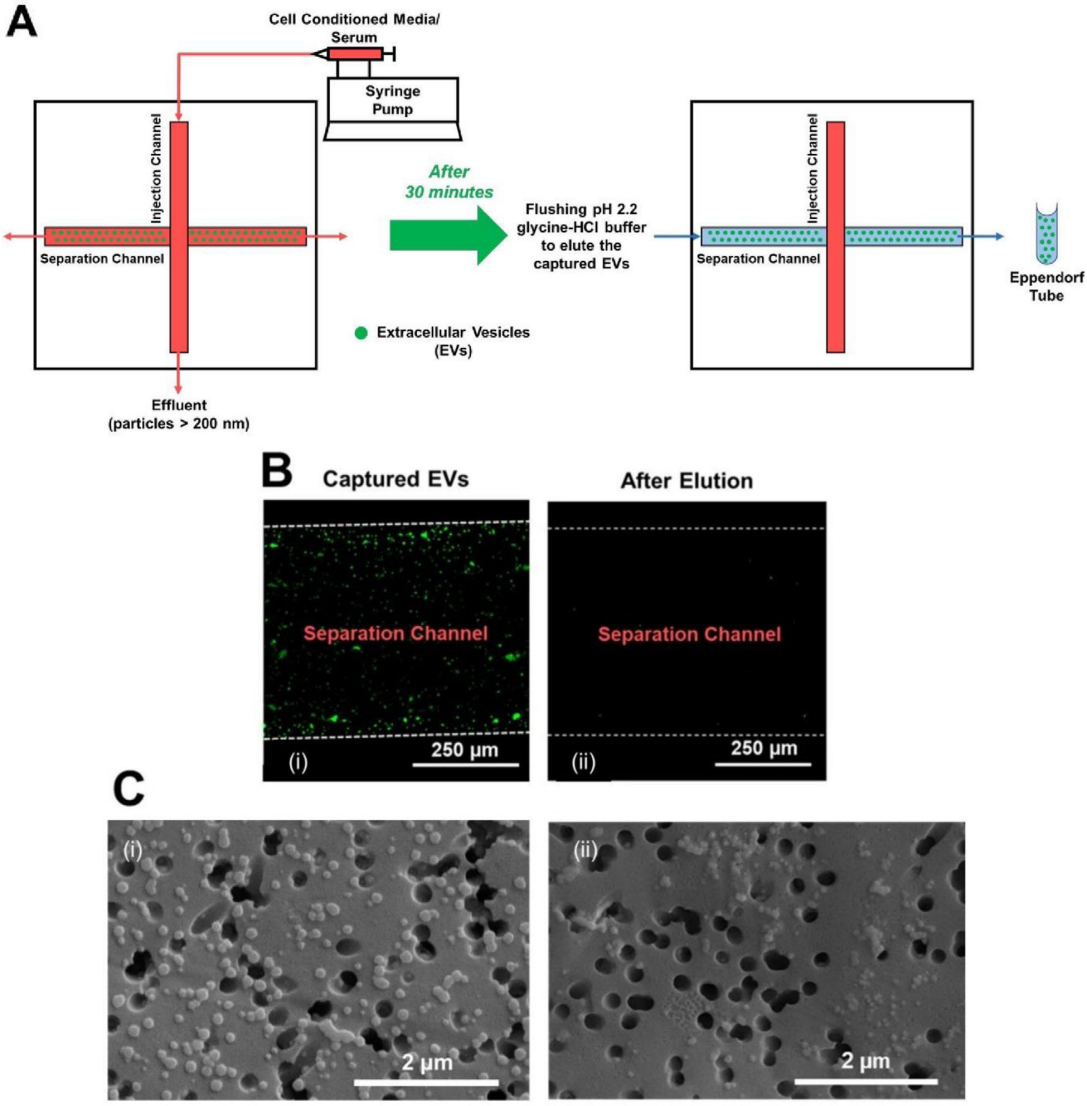
(A) Device schematic, depicting the experimental process of perfusing the cell conditioned media (LCCM) or serum. (B) Fluorescence verification for capture and elution of EVs: (a) Following passage of LCCM, the EVs tag to the anti‐CD63 antibody on the surface of the separation channel (green). The red arrow represents the direction of flow of LCCM in the separation channel; (b) Following elution with pH 2.2 glycine‐HCl buffer, there is a decrease in EV fluorescence (Little to no green). The blue arrow represents the direction of flow of glycine‐HCl buffer in the separation channel. (C) SEM image showing: (a) Representative images of LCCM derived EVs on the NCAM allowing visual inspection. EV diameter estimated to be between 125 nm and 165 nm; (b) DDLPS patient serum derived EVs on the NCAM allowing visual inspection. EV diameter estimated to be between 65 nm and 89 nm. We observe greater aggregation of DDLPS patient serum derived EVs compared to LCCM derived EVs

Figure 2A shows the schematic for the capture and elution of the isolated EVs. A falcon tube containing the LCCM at ‐80°C was thawed in a water bath at 37°C for 30 min. After thawing, the LCCM perfused through the injection channel of the micro‐nanofluidic device using a syringe pump (Harvard Apparatus, Model: 70–2213 Pico Plus). The LCCM containing syringe was connected to the device *via* a tubing (0.8 mm inner diameter; US Plastic) using barbed nylon connectors (Cole‐Parmer). The three remaining ports (outlet of the injection channel, and the inlet and outlet of the separation channel) were left open to atmosphere. The syringe pump was operated in the 10–25 μl/min range in increments of 5 μl/min for 30 min each with distinct devices to evaluate influence of injection flow rates on microfiltration and subsequent capture of the EVs.

Next, the separation channel was flushed five times with 1X PBS to rinse the channel and remove physiosorbed EVs and particles i.e., those not captured by the CD‐63 antibody immobilized within the separation channel. After the immunoaffinity capture of the EVs to the separation channel walls, the separation channel was flushed with 60 μl of pH 2.2 glycine‐HCl buffer to elute the captured EVs (Hisey et al., 2018) and the eluted sample was collected in an Eppendorf tube yielding sEV solution. Next, 120 μl of Tris‐HCl buffer was added to the Eppendorf tube to raise the pH of the glycine‐HCl buffer containing the EVs to ∼pH 7.4 and stored at ‐80°C until further use. Similar steps were repeated with DDLPS patient serum for isolation, capture, and elution of EVs to permit further analysis of the EVs and the cargo contained within the EVs. Unlike LCCM, instead of testing it for different flow rates, the DDLPS patient serum was perfused through the injection channel at a fixed flow rate of 10 μl/min for 30 min.

### 2.6 EV capture and release verification

In order to confirm if the EVs were indeed captured and subsequently released from the separation channel, DiO dye (ThermoFisher Scientific) was flushed through the separation channel with a 10‐min incubation at room temperature. The DiO tagging of EVs to the anti‐CD63 antibody was verified through confocal microscopy examination (A1R, Nikon) showing an increase in fluorescence in the separation channel (Figure 2B(i)). Following elution using the glycine‐HCl buffer the observed fluorescence decreased suggesting successful elution of the captured EVs as seen in Figure 2B(ii). Scanning electron microscopy (SEM) imaging showed not all EV particles passed through the NCAMs (Figures 2C(i) and 2C(ii)) despite being < 200 nm in size.

### 2.7 Numerical modelling

A computational model was developed to quantify the pressures driving microfiltration. For this numerical modelling a three‐dimensional (3D) computational domain was considered to quantify the EV isolation across the NCAMs and modelled using COMSOL Multiphysics (v.5.4). The computational domain models a smaller‐geometry than the entire device for reducing the computational burden. The injection channel was modelled as 1000 μm (L) x 250 μm (W) x 150 μm (H). A fully developed flow boundary condition at the inlet of the injection channel was imposed. The NCAM was modelled as a porous medium with the porosity, ε = 0.03 calculated on the manufacturer provided NCAM properties. All governing equations were solved under steady‐state, isothermal, and incompressible flow conditions (Rangharajan et al., 2016). The Navier‐Stokes equations were solved for the injection channel and the Brinkman equation was solved for the NCAM to account for membrane porosity and permeability using previously reported methods (Dehghani et al., 2019). The detailed description of the numerical model is available in the Supplementary Materials. Solutions from coarse meshes were re‐iterated with finer meshes until mesh‐insensitive solutions were achieved with a numerical tolerance of the converged solution at 10^–5^.

### 2.8 Ultracentrifugation and exoQuick isolation

The methods for state‐of‐art EV isolation using both ultracentrifugation and Exoquik‐TC have been reported extensively previously. Briefly, for ultracentrifugation isolation of LCCM derived EVs, protocol reported by Casadei and colleagues (Casadei et al., 2017) were followed. LCCM derived EVs were isolated using ExoQuick (System Biosciences), following the manufacturer's protocol.

### 2.9 Recovery rate estimation

The recovery rate defines the ratio of (EVs in the collection vial)/(EVs in the original LCCM or DDLPS patient serum). For the recovery rate, the total number of EVs was estimated for each collected volume from individual devices using nanoparticle tracking analysis (NTA; Malvern NanoSight NS300). The total NTA particle count was obtained by the cumulative count for each particle size measured in increments of 1 nm with the size range from 0.5 nm to 799.5 nm. Specifically, the recovery rate is a ratio of the total number of EV particles obtained from the sample to the total number of EV particles present in the LCCM that was passed through the device. As the flow rate was varied from 10 μl/min to 25 μl/min for 30 min, the obtained concentrations of particles through NTA analysis were obtained by adjusting for the respective volumes.

### 2.10 DNA isolation, quantification and sequencing

In order to quantify the EV cargo, Trizol LS was used for DNA extraction from the LCCM derived EVs eluted from the devices. DNA yield was determined using a Cytation3 reader (BioTek). *MDM2* DNA sequencing was analyzed by PCR amplification and subsequent DNA sequencing of exons 1, 6, and 10. PCR products were purified with the QIAquick PCR Purification Kit (Qiagen) according to the manufacturer's specifications. DNA sequencing was performed by the Genomic Shared Resource at the Ohio State University Comprehensive Cancer Center.

## 3 RESULTS

### 3.1 Capture efficiency of EVs depend on $Q_{\mathrm{IC}}$

**FIGURE 3:**
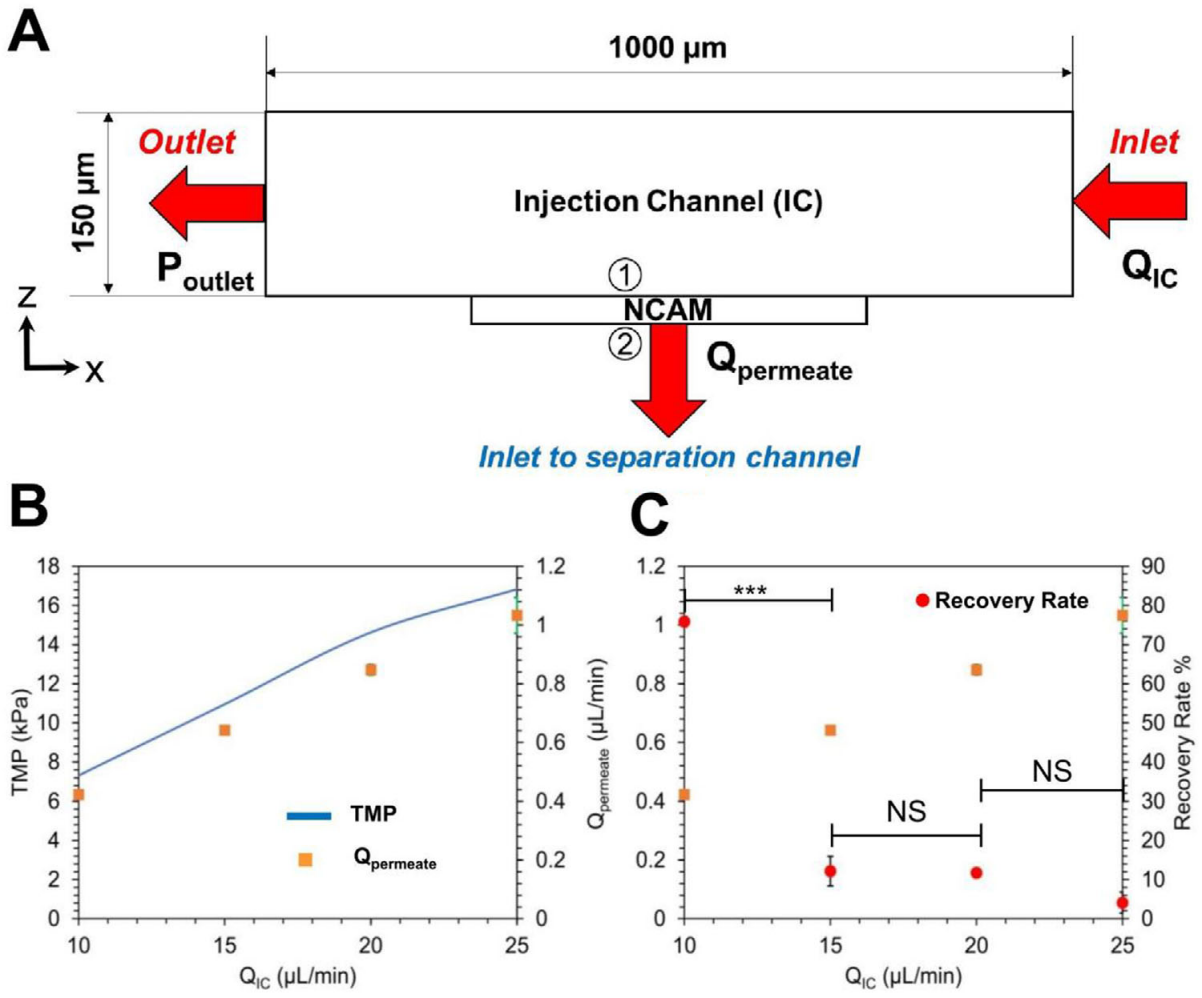
x‐z plane from the COMSOL model depicting all the boundary conditions and geometry of the modelling domain. (B) Variation in transmembrane pressure (TMP) and permeate flow rate (Qpermeate) for different injection channel flow rates (QIC = 10, 15, 20 and 25 μl/min). (C) Recovery rates in 30 min for different QIC (10, 15, 20 and 25 μl/min) and its corresponding Qpermeate (0.42, 0.64, 0.85 and 1.03 μl/min). * ‐ *P* < 0.05; ** ‐ *P* < 0.01;*** ‐ *P* < 0.001; NS – not significant. Error bars are ± Standard Error of the Mean

The micro‐nanofluidic device reported here conducts two distinct unit operations, first is the isolation of EVs using cross‐flow microfiltration and second an immunoaffinity capture. In order to capture most EVs in minimal time for the fixed device geometry, LCCM was perfused in the injection channel at volumetric flow rates ranging from 10 μl/min to 25 μl/min, in increments of 5 μl/min. For the cross‐flow filtration, the transmembrane pressure (TMP) across the NCAM was calculated using the 3D numerical model. The calculated TMP is the difference in pressures at the inlet (marked as 1) and outlet (marked as 2) of the NCAM (Figure 3A). Figure 3B shows that as $Q_{\mathrm{IC}}$ increased from 10 μl/min to 25 μl/min, the TMP also increased from 7.31 kPa ($Q_{\mathrm{IC}}$ = 10 μl/min) to 16.83 kPa ($Q_{\mathrm{IC}}$ = 25 μl/min). Interestingly, it was noted that over the 30 min of perfusion, approximately 4.2% of incoming fluid volume permeated across the NCAM from the inlet to the separation channel for capture of EVs regardless of the TMP (and therefore $Q_{\mathrm{IC}}$) as shown in Table S1.

Using NTA analysis, the capture efficiency of LCCM derived EVs for each flow rate was calculated. At 10 μl/min, a maximum recovery rate of 76% ± 1% was observed compared to EV recovery rates measured at higher flow rates: 15–25 μl/min, based on the differences in the measured averages from measurements on 3‐distinct microfluidic devices as described in Table S2. For flow rates ranging from 15–25 μl/min, recovery rates of less than 15% were observed (Figure 3C).

### 3.2 Isolation and capture of EVs from LCCM

**FIGURE 4:**
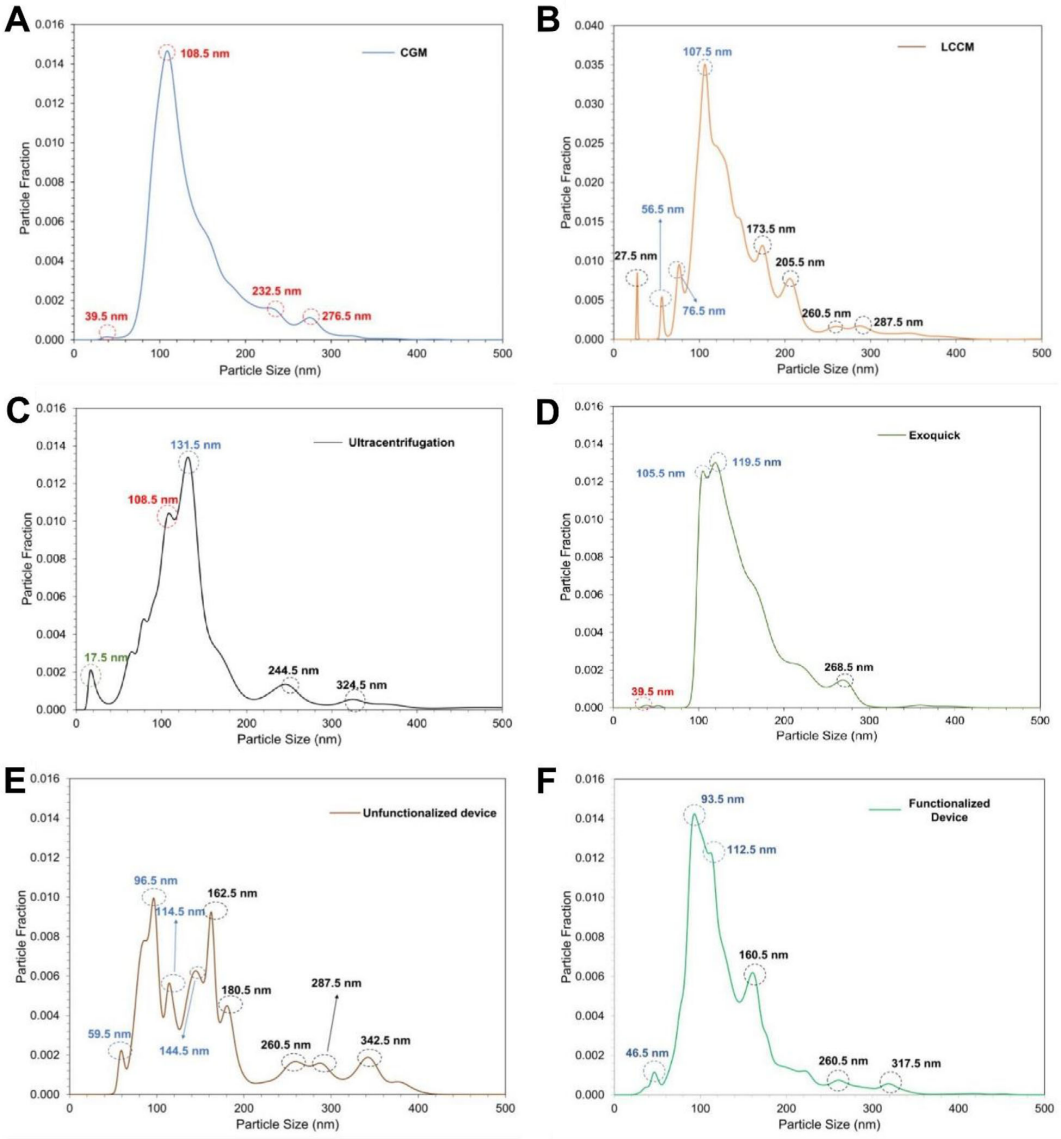
Size distribution according to the particle fraction obtained by NTA for (A) Cell Growth Media (CGM), (B) LCCM, (C) EV isolated using ultracentrifugation, (D) EV isolated using ExoQuick, (E) EV isolated using the micro‐nanofluidic device with the separation channel not functionalized with anti‐CD63 antibody and (F) EV isolated using the micro‐nanofluidic device with the separation channel functionalized with anti‐CD63 antibody. The peaks marked as dotted red circle represent the artifacts of the CGM. The peaks marked as dotted blue circle represent the sEV (30‐150 nm). The peaks marked as dotted black circle represents lEVs (100‐1000 nm) or EV aggregation seen as clumps in Figure 2C(i)

The size distribution of the LCCM‐derived EVs captured and eluted from the micro‐nanofluidic device was compared against both ultracentrifugation and ExoQuick‐based separations. The particle fraction of each size is shown in Figure 4 with the standard errors of mean for the major peaks in every measurement provided in Table S3. The particle fraction is the ratio of the number of particles of a certain size to the number of particles in the entire eluted sample. In order to establish a control for the particles contained within the media, we also measured the size distribution of particles present in the cell growth media (CGM) (Figure 4A) and the LCCM (Figure 4B). Peaks for particle sizes greater than 150 nm were noted for both the ultracentrifugation (Figure 4C) and ExoQuick‐based EV isolation (Figure 4D).

Previous work with microfluidic devices using size‐based separation has shown that collected EV solutions also contain particles other than EVs (Cho et al., 2016). Recently, it has been shown that immunoaffinity‐based techniques can be used to selectively capture exosomal population of EVs (Chen et al., 2010; Hisey et al., 2018; Kanwar et al., 2014) and therefore provide a higher purity of isolated EVs.

Here, we functionalized the separation channel with anti‐CD63 to capture EVs immediately after the size‐based isolation. Figures 4E and 4F compare the NTA particle size distributions without and with immunoaffinity‐based EV capture from our micro‐nanofluidic device. The size distribution of particles isolated using the non‐functionalized micro‐nanofluidic device showed multiple major peaks at 96.5 nm, 144.5 nm and 162.5 nm (Figure 4E). By contrast, the size distribution of the particles isolated using the functionalized micro‐nanofluidic device showed only two major peaks at 93.5 nm and 112.5 nm (Figure 4F).

### 3.3 LCCM derived EVs contain MDM2 DNA

**FIGURE 5:**
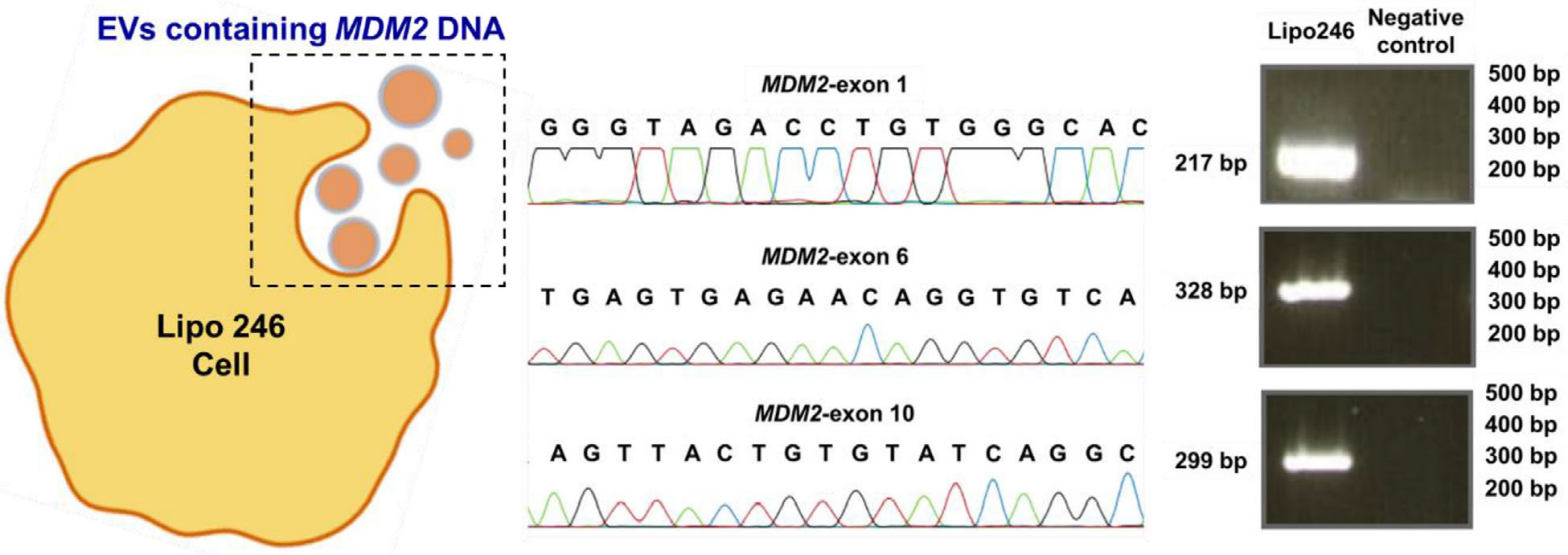
The schematic shows the EVs being released from cells relevant to DDLPS with the cargo of the EVs verified to contain MDM2 DNA by sequencing entire exons 1, 6 and 10. The red line represents Thymine (T), green line represents Adenine (A), blue line represents Cytosine (C), and the black line represents Guanine (G) ‐ the DNA base pairs (bp) expected in each exon were confirmed by a western blot analysis as shown

A key utility of EVs lies in the molecular cargo contained within the EVs, principally comprised of miRNA, mRNA, DNA, and proteins (Maas et al., 2017; Raposo & Stoorvogel, 2013). Moreover, past results have shown that the molecular content of the EVs can be damaged or yield poor purity (Cho et al., 2016; Davies et al., 2012) during the EV‐capture and lysis to access cargo. At the molecular level, DDLPS is characterized by *MDM2* gene amplification (Bill et al., 2016; Guan et al., 2015). Therefore, we examined *MDM2* DNA in LCCM‐derived EVs obtained by the isolation, capture, and subsequent elution of the EVs from the separation channel. DNA sequencing of the entire exons 1, 6, and 10 for *MDM2* not only demonstrated the presence of *MDM2* DNA within the captured and eluted EVs but also confirmed that this key DDLPS cargo was intact (Figure 5). Next, we estimated the total amount of DNA isolated from the EVs using the micro‐nanofluidic device and ExoQuick‐based separation. Using the micro‐nanofluidic device, we were able to isolate ∼27 ng/μl DNA (requiring 300 μl of LCCM and 30 min isolation time). In contrast, we were able to isolate only ∼5 ng/μl DNA from the EVs using ExoQuick technique and this required 30 ml of LCCM and 14 h isolation time. The DNA yield isolated from EVs using ultracentrifugation was lower than the one obtained with ExoQuick (∼2 ng/μl DNA from 30 ml of LCCM).

### 3.4 Isolation and capture of EVs from DDLPS patient serum

**FIGURE 6:**
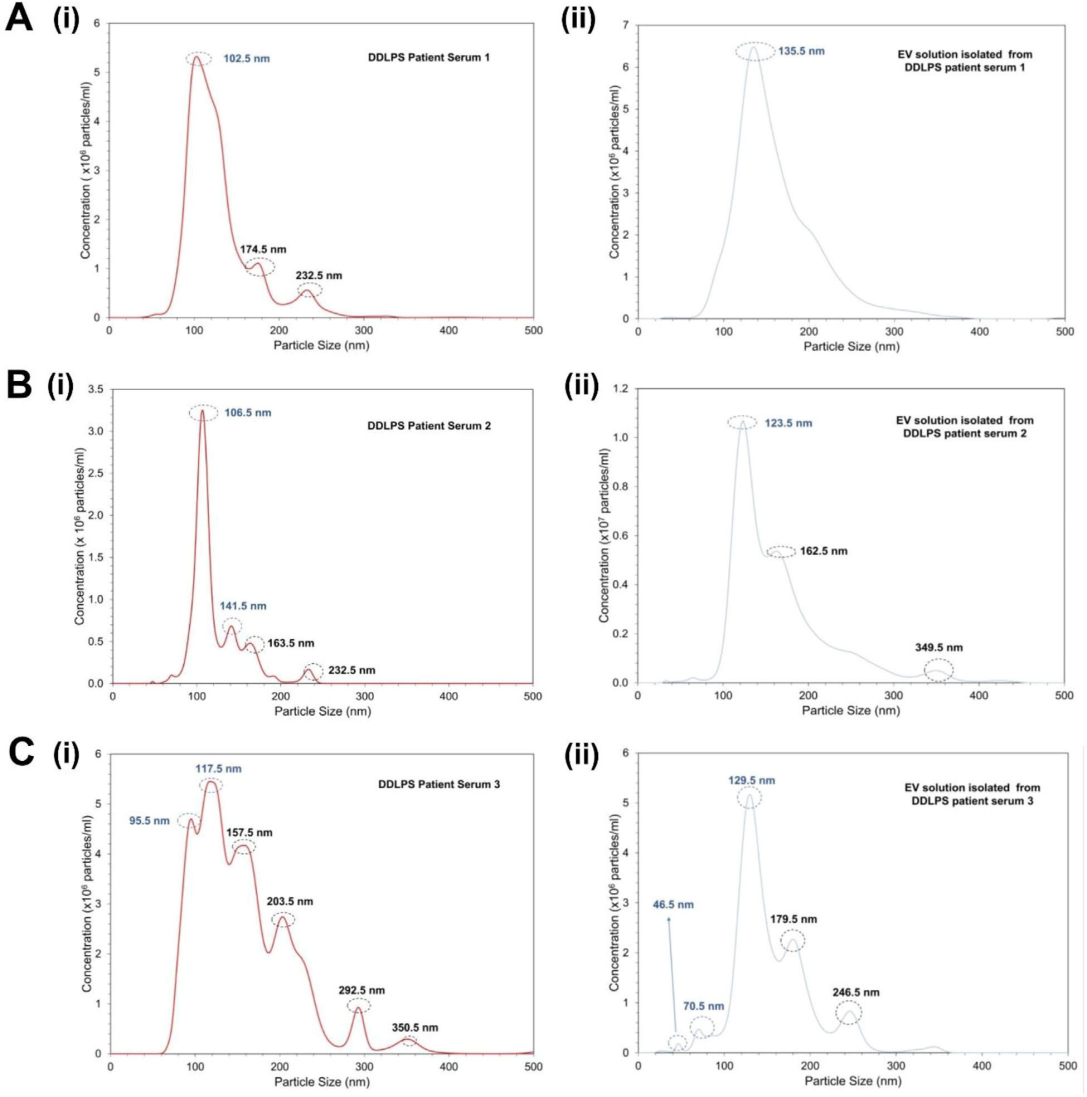
Size distribution obtained by NTA for (A) (i) DDLPS patient serum 1 and (ii) EV solution isolated from DDLPS patient serum 1 using the micro‐nanofluidic device. Size distribution obtained by NTA for (B) (i) DDLPS patient serum 2 and (ii) EV solution isolated from DDLPS patient serum 2 using the micro‐nanofluidic device. Size distribution obtained by NTA for (C) (i) DDLPS patient serum 3 and (ii) EV solution isolated from DDLPS patient serum 3 using the micro‐nanofluidic device. The peaks marked as dotted blue circle represent the sEV (30‐150 nm). The peaks marked as dotted black circle represents lEVs (100‐1000 nm) or EV aggregation seen as clumps in Figure 2C(ii)

Sera of five DDLPS patients were used to assess the viability for the possibility of extracting EVs from actual biological fluids using the micro‐nanofluidic device. In order to establish a control for the particles contained within the patient serum, we measured the size distribution of particles present in the serum based on the absolute concentration of particles of each size in the sample. The obtained size distribution of DDLPS patients is reported (Figures 6A(i), 6B(i) and 6C(i) with statistical context provided in Table S4). Additional patient data are also provided in Fig S1. Next, using the micro‐nanofluidic device we measured the size distribution of the particles in the EV isolated, captured, and eluted solution from DDLPS patient serum. As seen in Figure 6A(ii), the size distribution of the particles in the EV solution isolated from DDLPS patient serum 1 using the micro‐nanofluidic device shows a major peak at 135.5 nm measured by the NTA analysis. By contrast, multiple peaks were observed in the size distribution of the EV solution isolated from DDLPS patient serum 2 and 3 using the micro‐nanofluidic device (Figure 6B(ii), 6C(ii)). The difference in the isolation of EVs by this device is attributed to the heterogeneity inherent to patient sera and not due to an artefact of the EV isolation and capture process due to the microfluidic device, as reported in the discussion. Unlike for LCCM, where we recovered ∼ 76% of the EVs, for DDLPS patient serum, EV recovery was only ∼ 32% at the same injection channel flow rate of 10 μl/min and isolation time of 30 min.

## 4 DISCUSSION

In this work, we introduced a multi‐layer micro‐nanofluidic device to isolate, capture, and elute LPS‐derived EVs both from conditioned media and patient serum. Our micro‐nanofluidic device integrates two unit operations on the same device i.e., size‐based isolation using microfiltration and immunoaffinity‐based capture of EVs in a sequential manner allowing design, fabrication, and operation of the micro‐nanofluidic device compared to previous microfluidic‐based approaches (Cho et al., 2016; Davies et al., 2012; Lee et al., 2015; Wang et al., 2013).

A maximum recovery rate of 76% ± 1% for Q_IC_ = 10 μl/min was noted with the recovery rate decreasing to ∼15% with increase in $Q_{\mathrm{IC}}$ from 15 μl/min to 25 μl/min with differences at higher flow rates being within experimental uncertainty (Figure 3C). Therefore, for evaluating device operation patient sera, only the flow rate of 10 μl/min was used.

With the LCCM, the permeate flow rate through the NCAM was measured and used as input to a numerical model to calculate the TMP acting across the membrane at each $Q_{\mathrm{IC}}$. As the $Q_{\mathrm{IC}}$ increased from 10 μl/min to 25 μl/min, the corresponding TMP (7.31 kPa to 16.83 kPa) and $Q_{\mathrm{permeate}}$ (0.42 μl/min to 1.03 μl/min) also increased, as seen in Figure 3B. With increasing TMP, it would be expected that more EVs will permeate across the NCAM to the separation channel for subsequent capture. However, as the TMP increased, the EV recovery rate dropped (Figure 3C). Previously, Dehghani *et al*. (Dehghani et al., 2019) reported a microfluidic device that captured EVs on the membrane and allowed proteins to pass through under an applied pressure difference. They showed that thin membranes (< 1 μm) were better suited than track‐etched membranes (6‐10 μm thick), since the thicker membranes required higher trans‐membrane pressure (TMP) to drive the EVs through the membrane, which in turn caused the pores of the track‐etched membranes to be clogged by the EVs due to membrane fouling.

In the immunoaffinity EV capture, a critical step is the binding between the EV and the tethered antibody. Moreover, past works have reported a finite residence time to allow adequate capture of EVs on the functionalized substrate. In previous work utilizing antibodies for CD9, CD63, and CD81 to capture the EVs, the residence time of these EVs have been shown to vary from 5 min to 40 min (Chen et al., 2010; Hisey et al., 2018; Kanwar et al., 2014; Zhang et al., 2019). Here, for the separation channel volume of 2.25 μl, the residence time for EVs in contact with the antibody tethered to the functionalized separation channel decreased from 5.3 min at $Q_{\mathrm{IC}}$ = 10 μl/min to 2.2 min at $Q_{\mathrm{IC}}$ = 25 μl/min. We note that the residence time of 5.3 min (at 10 μl/min) was found to yield the highest capture‐release EV efficiency, which is near the lower end of the previously reported residence times (Kanwar et al., 2014).

A review of the past results on the immunoaffinity‐based microfluidic devices for EV capture also suggests a complex role for underlying fluid mechanics. These past microfluidic devices typically tend to operate in the high Peclet number, Pe (which is the ratio of advective flow to diffusive flow) regime of O(10^4^) (Chen et al., 2010; Kanwar et al., 2014). As Pe > > 1, the binding of EVs to the antibody will be dominated by advection of the EVs in the devices. Therefore, as the volumetric flow rate ($Q_{\mathrm{permeate}}$) increases in the separation channel and residence time declines, fewer EVs should be captured. Indeed, compared to 10 μl/min the higher flow rates demonstrated a lower rate of EV capture (Figure 3C). The Pe for the micro‐nanofluidic device was 6.77 × 10^3^ (at $Q_{\mathrm{IC}}$ = 10 μl/min and $Q_{\mathrm{permeate}}\;$= 0.42 μl/min) and 1.64 × 10^4^ (at $Q_{\mathrm{IC}}$ = 25 μl/min and $Q_{\mathrm{permeate}}$ = 1.03 μl/min). While there were differences in captured EVs at 15–25 μl/min cases (Figure 3C), the differences cannot be resolved beyond experimental uncertainty. We did not evaluate the effect of microchannel dimensions and aspect ratio on the capture efficiency and as such a parametric study is beyond the scope of this paper. A complete analysis for different TMP due to systematically varied flow rates and channel dimensions will be the engineering path to optimizing micro‐nanofluidic device design and operation for isolation and selective capture of the isolated EVs. Therefore, the flow rate dependence and the reduction in the EV recovery shows that isolation of EVs using size‐based filtration is a complex fluid mechanics problem with multiple parameters to consider for optimal EV isolation.

We note that this work compares multiple methods for EV isolation and extraction of LPS‐relevant EV‐cargo. For example, UC is operated through a multiple step process starting at an acceleration of 300 g and reaching a maximum of 10,000 g. Our microfluidic devices by contrast operate in a creeping flow configuration (Lochab & Prakash, 2020) with particle flow in the viscous flow regime. Similarly, the ExoQuick is a precipitation based method with minimal flow. While the underlying fluid properties likely remain similar across methods, the processing of these fluids to yield measurable differences in EVs (as shown in Figure 4) and subsequent EV‐cargo were compared across the various methods.

Figure 4 shows a summary of the size distribution of EVs captured from various media with different methods. All measurements were done in triplicate with three different media samples. Measurements on the microfluidic devices were done with three distinct devices.

Figure 4A shows that the dominant particle size isolated from the cell growth media (CGM) was 108.5 nm and that from LCCM was 107.5 nm (Figure 4B). Interestingly, Figure 4B also shows several additional particle sizes ranging from 27.5 nm – 286.5 nm, with the particles at 118.5 nm and 173.5 nm forming a major fraction of the total sample.

Figure 4C and Figure 4D show the results from isolation of EVs by using ultracentrifugation (UC) and Exoquick based precipitation for the LCCM. The dominant peaks for UC were at 108.5 nm and 131.5 nm. Notably, the 108.5 nm peak is similar to the CGM peak in Figure 4A, albeit at a lower particle fraction. Exoquick precipitation led to identifiable peaks at 105.5 nm and 119.5 nm. Figures 4E and 4F capture the performance of the microfluidic device with the 2‐stage isolation (through NCAM) and the immunoaffinity capture with the antibody tag (Figure 4F). As seen in Figure 4E, multiple particle sizes were isolated ranging from 59.5 nm – 342.5 nm with the most dominant size identified at 96.5 nm. After isolation the use of the CD‐63 antibody to capture a subset of EVs yielded fewer peaks ranging from 46.5 nm – 317.5 nm, with the dominant peaks at 93.5 nm, 112.5 nm, and 160.5 nm. Clearly, the CGM and LCCM contain particles with many different sizes and the various methods show that this variability is preserved in the isolation of the EVs from these solutions.

We observed a peak at 108.5 nm (marked as a dotted red circle) in NTA‐quantified size distributions, this peak is present in the broad distribution of both the Exoquick (105.5 nm‐ 119.5 nm) and LCCM (107.5‐118.5) (Figure 4D) which arises from the cell growth media (CGM) that was used for culturing the liposarcoma cells (Figure 4A). The same peak seems to be present within a broad distribution of other particles (107.5‐118.5) in the NTA from the LCCM. Interestingly, this peak at 108.5 nm was also observed when we isolated the EVs without immunoaffinity capture in the separation channel (Figure 4E). However, the size distribution seen for the EVs isolated and captured using the microfluidic device with the immunoaffinity capture in separation channel did not have the peak at 108.5 nm (Figure 4F). Collectively, these results suggest that even though the peak at 108.5 nm may be in the size range for EVs, the particles are likely not relevant for the EVs since the immunoaffinity tags did not capture these particles. Moreover, the unfunctionalized device show a not selective capture (Figure 4E), while the functionalized device showed fewer peaks (Figure 4F), showing that we were selectively capturing more sEV by tagging them to anti‐CD63 antibodies present in the separation channel. Such selective capture may be a desirable outcome to obtain higher purity of the EVs for subsequent extraction of EV cargo. However, as this work focuses on demonstrating the viability of a micro‐nanofluidic device for isolation and capture of EVs for extraction of liposarcoma specific biomarker, a detailed analysis on the purity of captured EVs was not performed and remains an open question. Furthermore, the SEM images of the EVs revealed that the size ranges from 125 nm to 165 nm; this falls within the previously reported size range for EVs (Klinke et al., 2014; Théry et al., 2009) (Figure 2C(i)). Additionally, no particle size less than 30 nm were observed for our device, suggesting a lack of protein aggregation. In contrast, for ultracentrifugation, peaks at particle size < 30 nm suggested the aggregation of proteins, as previously reported (Momen‐Heravi et al., 2013).

The purity of EVs is important for eventual analysis of EV‐contained cargo. Previously, Davies et al. developed a microfluidic filtration device comprising a photo‐patterned porous polymer monolith membrane to isolate EVs from blood (Davies et al., 2012). The device used a sample volume of 240 μl but showed limitations like formation of gas bubbles near electrodes, fabrication complexities associated with the isolation membrane, and lower purity RNA compared to ultracentrifugation. Cho et al. (Cho et al., 2016) used electromigration to isolate EVs. During the migration, a track etched polycarbonate membrane with 30 nm pores captured biomolecules with diameters larger than the pore size while allowing biomolecules smaller than the pore size to pass through, thereby isolating the EVs onto the membrane. Their method allowed a relatively fast EVs isolation (∼30 min) with a 65% EV recovery. Even though their system was able to operate 10X faster and recover 14X more EVs compared to ultracentrifugation, the purity of the isolated EVs was lower compared to ultracentrifugation.

The peaks at 223.5 nm and 317.5 nm for our device (Figure 4F) suggests that the larger particles within LCCM and CGM may not be entirely filtered out or particles aggregate after permeating through the NCAMs. As a possible explanation on how larger particles can traverse the pores, we note that NCAMs are prepared by chemical etching following a nuclear track‐etch process (W. P., 2016). The nominal size of the nanocapillaries is 200 nm; however, per the manufacturer, a variation of +/‐ 10%–15% in size can be expected (Vitarelli Jr et al., 2011).

The use of NCAMs for various microfluidic devices and distribution of pores is well‐known and has been reported previously (Vitarelli Jr et al., 2011). Previous reports with membrane‐based isolation of vesicles have also reported membrane‐bending causing different types of vesicles to pass through the membrane (Doskocz et al., 2020). Therefore, with the reported pore size distributions and past reports there are many likely reasons for observing some EVs larger than the 200 nm nominal size.

Importantly, we can observe in Figures 4 and 6 that the sizes of EVs harvested by the device from cell culture media and from serum range from 46.5 nm – 350.5 nm, so both types of EVs (sEVs and lEVs) are present in the samples. While the importance of sEVs have been largely studied, lEVs are also emerging as containing important cargos and potentially useful for cancer biomarker discovery (Ciardiello et al., 2020; Salem et al., 2020). Here we show that the EVs isolated from the micro‐nanofluidic device contain both sEVs and lEVs and the MDM2 was obtained from this mix of sEVs and lEVs.

One of the molecular hallmarks of dedifferentiated liposarcoma is high levels of *MDM2* DNA, a finding observed in nearly all DDLPS tumors (Guan et al., 2015). Casadei *et al*. (Casadei et al., 2019) have previously shown the presence of *MDM2* DNA in EVs isolated both from the tumor and from the patient circulation. They demonstrated that in light of this cargo, DDLPS EVs may play an important role in DDLPS loco‐regional metastasis. Past results for microfluidic assays analyzing integrity of EV cargo have reported variable results in maintaining the integrity of the EV contents (Cho et al., 2016; Davies et al., 2012). Importantly, when we analysed the vesicle contents of the particles obtained with this device, we showed that we are able to obtain the same results as analysis of the content of vesicles isolated by ultracentrifugation (Figure 5). After micro‐nanofluidic isolation, capture, and elution of the vesicles and subsequent Sanger Sequencing of the entire exons 1, 6, 10 of *MDM2* (using primers built on the introns before and after each exon; primer descriptions in Casadei *et al*. (Casadei et al., 2019) we found the presence of MDM2 DNA within isolated EVs. A key implication of this finding is that the isolation, capture, and elution of the EVs did not compromise the internal cargo that is a critical biomarker for DDLPS. Furthermore, this result also demonstrates that the output of this device is comparable to ultracentrifugation, suggesting that the two methods align with regards to vesicle isolation and also vesicle content. This key finding is being further explored by our group in a clinical trial examining *MDM2* DNA of DDLPS derived EVs as a potential prognostic and predictive biomarker using the micro‐nanofluidic device. These findings suggest many new and important potential applications for this device in a point‐of‐care setting. Such usage will require significant optimization of the various parameters reported here which are essential to reliable operation of micro‐nanofluidic devices; these studies are already underway by our group, seeking to overcome the comparative limitations of both ultracentrifugation and ExoQuick methods. Furthermore, the identification of this key target in the EVs isolated using our device shows that the EVs contain the valuable MDM2 cargo relevant for LPS.

With the goal of point‐of‐care use, we evaluated isolation and capture of EVs from five different DDLPS patient blood sera. The size distribution of the particles in the EV solution isolated from DDLPS patient serum using the micro‐nanofluidic device (Figures 6A(ii), 6B(ii), and 6C(ii)) were within with the size range of the EVs (major peaks at135, 123, 129 nm) and also visually confirmed by the SEM images (Figure 2C(ii)). The micro‐nanofluidic device enabled recovery of ∼9.3× 10^7^ EVs from DDLPS patient 1 serum and ∼1.3× 10^8^ EVs from DDLPS patient 2 serum and ∼5.7× 10^7^ EVs from DDLPS patient 3. Past reports have shown that these levels of isolated and captured EVs are suitable for performing extensive functional *in vitro* assays (requiring ∼10^10^ EVs/10^6^ cells) and therapeutic *in vivo* studies (requiring ∼2 × 10^10^ EVs/mouse) (Cavallari et al., 2017; Haga et al., 2017).

For patient serum results, the device was operated at 10 μl/min injection flow rate that gave ∼76% recovery rate for LCCM, we observed only a ∼32% EV recovery rate. EVs that were captured in the separation channel primarily rely on the immunoaffinity tagging due to the CD63 marker and therefore use only one capture point from a possible range of capture sites. Moreover, as shown in Figure 3B, the immunoaffinity capture is impacted by flow conditions. This low recovery rate could also be attributed to the complexity of the serum as compared to the conditioned media. The heterogeneity observed in EV size distribution from the serum samples reflects the heterogeneity of this disease (Matthyssens et al., 2015). Tumor heterogeneity is also presented at the tissue level and has been reported for other types of cancer with 40% or higher variation in tumor properties reported between patients (Lochab et al., 2020; Prakash et al., 2015). In order to obtain insights to the heterogeneous size distributions a comparison of the difference between NTA of the same serum sample before and after the device rather than comparison of the sera samples across patients was reported in Figure 6.

In summary, this is the first report demonstrating the successful isolation, capture, and elution of liposarcoma‐derived EVs using a micro‐nanofluidic device. We were able to drop the processing time by almost 85% to ∼ 30 min compared to ultracentrifugation (3‐4 h) and 94% compared to ExoQuick (∼12 h). In a detailed characterization, the dependence of EV capture efficiency on the injection channel flow rate was reported with a maximum EV recovery rate of 76 84% ± 21% at 10 μl/min injection flow rate due to the residence time available for the EVs to bind to the CD‐63 antibody tags. Moreover, the EV isolation and capture process did not damage the critical MDM2 DNA cargo from the EVs, but we were able to reduce the needed sample volume and time significantly. Analysis of the captured and subsequently eluted (sEV ‐enriched population of) EVs showed a nearly five‐fold increase in the amount of DNA isolated from the micro‐nanofluidic device compared to EVs isolated using ExoQuick. In the context of a potential point‐of‐care use for micro‐nanofluidic diagnostics, using less sample volume for higher yield is beneficial as it may permit additional analysis from available biofluids. The integrity of the EV cargo was verified by confirming the DNA sequence of *MDM2* with potential viability of the device for clinical use demonstrated by recovering ∼ 10^10^ EVs from 300 μl DDLPS patient serum. Furthermore, we report data from not only the capture of EVs from conditioned media but also from blood sera of 5 distinct patients to demonstrate the incredible heterogeneity of these tumors. The results and the underlying parameters studied here show the complexity of isolating, capturing, and eluting EVs for disease‐specific biomarkers while reporting on a possible path to optimizing device operation for potential clinical or point‐of‐care setting use.

## CONFLICTS OF INTEREST

The authors report no conflicts or competing interests to declare for the work reported in this paper.

## Supporting information

Supporting Information
