# Supplementary material for: Cross‐flow microfiltration for isolation, selective capture and release of liposarcoma extracellular vesicles
Source: J Extracell Vesicles. 2021 Feb 16;10(4):e12062. doi: 10.1002/jev2.12062 (PMC7887429; doi:10.1002/jev2.12062)
Supplement: Supplementary file 1 — Supporting Information [file JEV2-10-e12062-s001.docx]

**Supplementary Material**

**Computational Domain:**

The computational domain was the injection channel and the NCAM inlet to the separation channel. Due to the large size and extensive computational resources needed to model the entire device, a smaller section of the injection channel was used with dimensions 1000 µm x 250 µm x 150 µm, capturing the essential fluid mechanics using a methodological approach used previously for efficient numerical calculations^1^. The NCAM dimensions used were 500 µm x 250 µm x 10 µm, capturing the actual thickness of the NCAM as provided by the manufacturer.

**Governing Equations:**

COMSOL Multiphysics was used to solve the governing equations for standard porous-flow fluid mechanics. The fluid flow in the injection channel is governed by the Navier-Stokes equations^2^ while the flow through the porous membrane was modeled by the Brinkman equations^3^. All the equations were solved for steady, incompressible, and isothermal conditions. The Navier-Stokes equations with the continuity equation are:

$\text{ρ(u}\cdot\nabla u\text{) = -}\nabla\text{p + }\nabla\cdot\text{(}\eta\text{(}\nabla\text{u+(}\nabla\text{u)}^{T}))$,

.

Where, ρ is the density of the fluid in kg/m^3^, u is the velocity of the fluid in m/s, p is the pressure in Pa, and η is the dynamic viscosity in Pa.s. The Brinkman equation was used as:

$-\nabla\cdot\frac{\eta}{\varepsilon}\left(\nabla u+\left(\nabla u\right)^{T}\right)-\left(\frac{\eta}{\kappa}u+\nabla p\right)=0$.

Where, η is the dynamic viscosity in Pa.s, ε is the porosity of the membrane, u is the velocity of the fluid in m/s, κ is the permeability of the membrane in m^2^, and p is the pressure in Pa. The porosity of the membrane was obtained from the manufacturer provided nominal pore size, pore density, and membrane area and does not account for the variation in these parameters in the computational model^4^. The permeability was calculated from the Darcy’s coefficient of permeability^5^ in which the flow rate and pressure drop across the membrane was replaced with pore resistance^6^ and is found to be 3.9 x 10^-17^ m^2^.

**Boundary Conditions:**

A fully developed, laminar flow with a constant flow rate was imposed at the inlet of the injection channel while the outlet of the injection channel was assigned a constant pressure open to the atmosphere to approximate realistic device operation. The flow rate through the outlet of the membrane (Q_permeate_) was assigned a constant value using experimentally determined volume of liquid collected through the separation channel. The injection channel flow rates and the corresponding flow rates from membrane are listed in Table S1.

**Table S1.** Summary of injection channel flow rate and corresponding flow rate through membrane

| **Q_IC_** (µl/min) | **Q_permeate_** (µl/min) |
| --- | --- |
| 10 | 0.42 |
| 15 | 0.64 |
| 20 | 0.85 |
| 25 | 1.03 |

The walls of the injection channel were assumed to be impermeable with the no-slip boundary condition. Similar boundary conditions were also imposed on the walls of the membrane.

**Supplementary Data Tables**

**Table S2*.*** Summary of the recovery rates for different flow rates in injection channel with the corresponding standard errors (Figure 3b; experimental data).

| **Q_IC_** (µl/min) | **Recovery Rate** | **Standard Error of the Mean** |
| --- | --- | --- |
| 10 | 75.9 | 1.34 |
| 15 | 12.2 | 3.77 |
| 20 | 11.7 | 1.20 |
| 25 | 4.1 | 2.69 |

**Table S3.** Summary of the major peaks identified in Figure 4 showing the peak location from the NTA analysis, particle fraction at each major size peak and the associated standard error in the mean arising from multiple measurements.

| **Measurement** | **Peak (nm)** | **Particle Fraction** | **Standard Error of the Mean** |
| --- | --- | --- | --- |
| CGM | 108.5 | 0.015 | 0.0034 |
| LCCM | 107.5 | 0.035 | 0.0042 |
| Ultracentrifugation | 131.5 | 0.013 | 0.0036 |
| Exoquick | 119.5 | 0.013 | 0.0043 |
| Unfunctionalized Device | 96.5 | 0.001 | 0.0023 |
| Functionalized Device | 93.5 | 0.014 | 0.0038 |

**Table S4.** Summary of the key peaks identified in Figure 6 showing the peak location from the NTA analysis, concentration in particles/ml at each identified size peak, and the associated standard error in the mean arising from multiple measurements.

| **Patient number** | **Sample composition** | **Peak** | **Concentration (particles/ml)** | **Standard Error of Mean (particles/ml)** |
| --- | --- | --- | --- | --- |
| 1 | Serum | 102.5 nm | 5.32 | 0.22 |
|  | Device | 135.5 nm | 6.48 | 2.40 |
| 2 | Serum | 106.5 nm | 3.25 | 0.87 |
|  | Device | 123.5 nm | 1.07 | 0.09 |
| 3 | Serum | 117.5 nm | 5.45 | 0.83 |
|  | Device | 129.5 nm | 5.17 | 1.57 |

**References:**

1. K. K. Rangharajan, M. Fuest, A. Conlisk and S. Prakash, Microfluidics and Nanofluidics **20** (4), 54 (2016).

2. F. M. White, *Fluid Mechanics*. (McGraw- Hill, New York, 2011).

3. H. C. Brinkman, Applied Science Research, **1**, 81 (1949).

4. K. Kim, P. V. Stevens. A.G. Fane, Journal of Membrane Science, **93** (1), 79 – 92 (1994).

5. H. E. Pacella, H. J. Eash, W. J. Federspiel, Journal of Membrane Science **382**(1-2), 238-242 (2011).

6. M. Dehghani, K. Lucas, J. Flax, J. McGrath and T. Gaborski, Advanced Materials Technologies **4** (11), 1900539 (2019).

**Supplementary Figures**

**Figure S1**

**
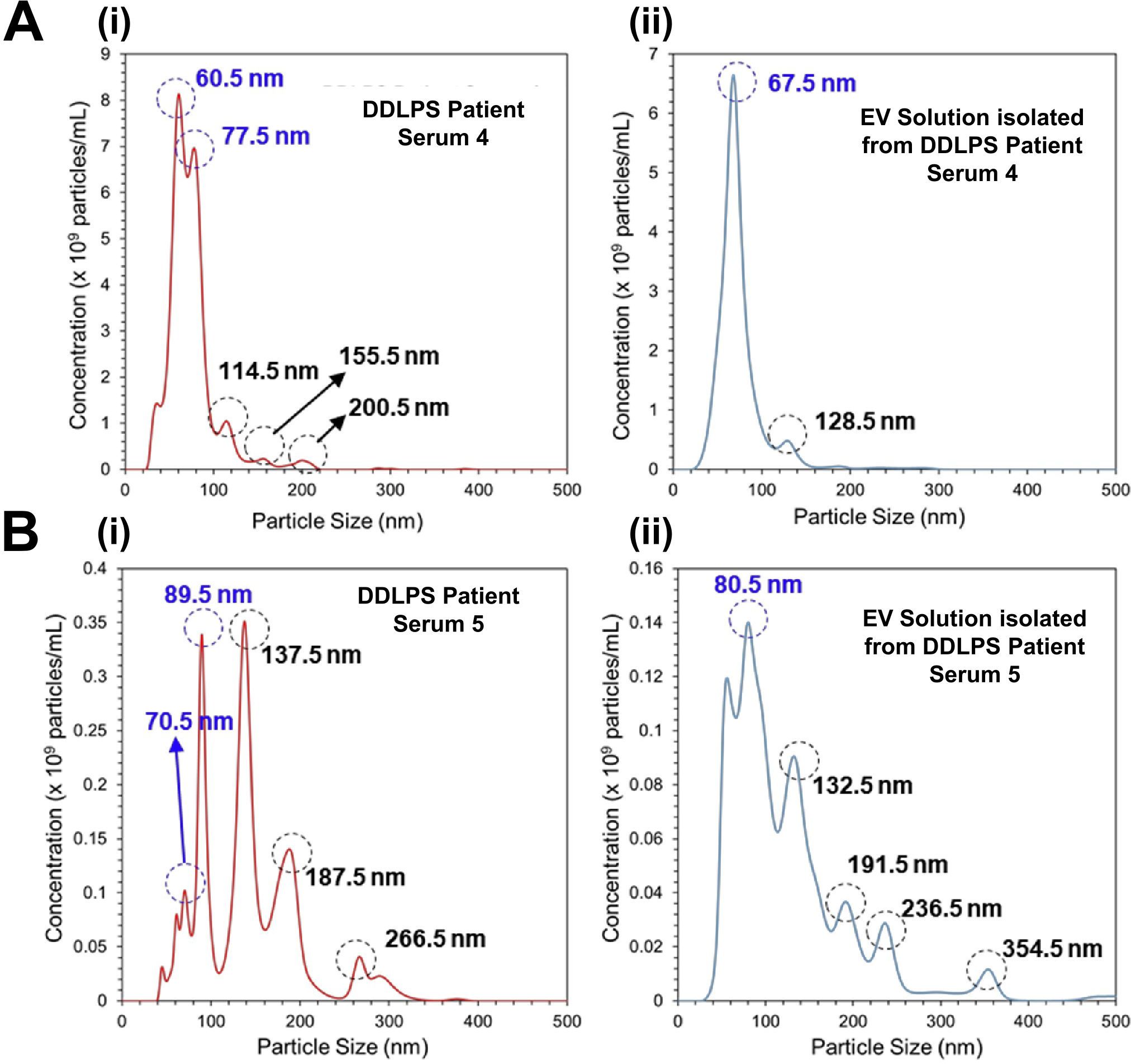
**

**Figure S1:** Size distribution obtained by NTA for (A) (i) DDLPS patient serum 4 and (ii) EV solution isolated from DDLPS patient serum using the microfluid device. Size distribution obtained by NTA for (B) (i) DDLPS patient serum 5 and (ii) EV solution isolated from DDLPS patient serum 5 using the microfluid device. The peaks marked as dotted blue circle represent the sEV population (30-150 nm). The peaks marked as dotted black circle represent lEVs (100-1000 nm) or EV aggregation seen as clumps in Figure 2C (ii).
